# Effect of Unemployment and the Dole on the Health and Habit of the Nation

**Published:** 1923-01

**Authors:** Olga Nethersole

**Affiliations:** Honorary Organiser, The People's League of Health


### Effect of Unemployment and the Dole on the Health and Habit of the Nation.

*To the Editor of* The Hospital and Health Review.

Dear Sir,—In view of the ever-increasing need of inquiry into the cause of, and legislation for, the disease of unemployment and its consequent suffering and danger to our well-being as a nation, I venture to enclose the printed verbatim report of the deputation from the People's League of Health to the Minister of Health on July 6th last on the subject of the "Effect of Unemployment and the Dole on the Health and Habit of the Nation."

The speakers were members of the Medical and Lay Councils of the League, who gave their expert knowledge and considered opinions on the subject and made many practical suggestions which I would ask you to kindly peruse, and if they meet with your approval, back them up in the columns of your valuable newspaper, insisting on the need for an inquiry to root causes, rather than palliative measures for symptoms of the disease.

Sincerely I sign myself,
Olga Nethersole,
Honorary Organiser, The People's League of Health.

### The Dentists' Register.

*To the Editor of* The Hospital and Health Review.

